# Advances in Molecular and Translational Medicine

**DOI:** 10.3390/ijms24097726

**Published:** 2023-04-23

**Authors:** Mariarosaria Boccellino

**Affiliations:** Department of Precision Medicine, University of Campania “Luigi Vanvitelli”, 80138 Naples, Italy; mariarosaria.boccellino@unicampania.it

Translational medicine is an interdisciplinary field that combines basic research findings with clinical practice to accelerate the development of new diagnostic tools, therapies, and preventive strategies for human diseases [1]. This approach aims to bridge the gap between laboratory discoveries and their application in clinical settings, with the ultimate goal of improving patient outcomes [2,3]. By studying the molecular and cellular mechanisms underlying complex diseases, translational medicine seeks to develop targeted treatments that are personalized relative to the individual patient’s needs [4,5]. Via close collaboration between basic scientists, clinical researchers, and healthcare professionals, translational medicine is driving the development of innovative therapies and improving our understanding of disease pathogenesis [6,7].

The Special Issue “Advances in Molecular and Translational Medicine” aimed to invite worldwide investigators and clinicians confident in translational medicine research to submit their most interesting research in the field of molecular and translational medicine applications.

Delcroix, V. et al. discuss the role of inflammasome activation in lacrimal gland inflammation and its association with dry eye disease and Sjögren’s syndrome. The study used various models of inflammation, including bacterial infection, acute injury, and chronic inflammation in mouse lacrimal glands. These included bacterial infection (mimicked by the injection of lipopolysaccharide and nigericin), acute injury (induced by interleukin-1α injection), and chronic inflammation (studied in two Sjögren’s syndrome models: diseased *NOD.H2*^b^ compared to healthy *BALBc* mice and Thrombospondin-1-null compared to TSP-1^WT^ *C57BL/6J* mice). The study found that inflammasomes were activated in epithelial cells in all models of inflammation, leading to the upregulation of various immune response genes such as caspases and interleukins Il1b and Il18. The authors also observed increased IL-1β maturation in Sjögren’s syndrome models compared to healthy control lacrimal glands. The study also investigated the role of lipid metabolism in lacrimal gland inflammation. Using the RNA-seq data of regenerating lacrimal glands, the authors found that lipogenic genes were upregulated during the resolution of inflammation following acute injury. However, in chronically inflamed *NOD.H2*^b^ lacrimal glands, altered lipid metabolism was associated with disease progression. Specifically, genes involved in cholesterol metabolism were upregulated, while genes involved in mitochondrial metabolism and fatty acid synthesis were downregulated, including peroxisome proliferator-activated receptor alpha (PPARα)/sterol regulatory element-binding 1 (SREBP-1)-dependent signaling. The findings suggest that targeting inflammasome activation and restoring lipid metabolism may be potential therapeutic strategies for treating dry eye disease and Sjögren’s syndrome [8]. The study by Zuccato, C. investigated the role of the LYAR protein and its polymorphism rs368698783 (G > A) in regulating fetal hemoglobin (HbF) production in erythroid precursor cells (ErPCs) of β-thalassemia patients. The LYAR protein was found to be a potential regulator of γ-globin gene transcription, with its DNA-binding motif located within the 5^′^-UTR of the Aγ-globin gene. The rs368698783 polymorphism decreased the LYAR binding efficiency to the Aγ-globin gene. The study found that rs368698783 polymorphism was associated with high basal contents and induced the production of HbF in β-thalassemia patients. In particular, the most significant association was found when rapamycin was used as an HbF inducer. The study suggests that the rs368698783 polymorphism could be a useful parameter for identifying β-thalassemia patients with a high probability of responding to in vivo hydroxyurea (HU) treatment. These findings have potential implications for both basic biomedicine and applied translational research for personalized therapy in β-thalassemia [9]. In the study by Feige et al., a relationship has been highlighted between common diseases such as diabetes mellitus and acute hyperglycemia, which can increase myocardial infarction (MI) morbidity and mortality. The study found that preconditioning with transferred remote ischemic preconditioning (RIPC) plasma under normoglycemia significantly reduced infarct size compared to control hearts. Under acute hyperglycemia, applying RIPC plasma led to a significant reduction in infarct size compared to control hearts, but the protective effect was weaker than under normoglycemia. Prolonged hyperglycemia completely abolished the cardioprotective effect of RIPC plasma. These findings suggest that acute hyperglycemia does not completely hinder the cardioprotective effects of RIPC plasma, but prolonged hyperglycemia does [10]. In a study that aimed to investigate the role of tumor-associated macrophages (TAMs) and programmed death ligand 1 (PD-L1) in breast cancer progression and response to therapy, high-throughput multiple immunohistochemistry was used on 81 breast cancer samples. The results showed that the triple-negative subtype of breast cancer had significantly more CD68/CD163, CD68/PD-L1, and CD163/PD-L1 double-positive cells than the estrogen receptor (ER)/progesterone receptor (PR) subtype in both stromal and intratumoral areas. Moreover, it was also found that a higher number of CD68^+^/PD-L1^+^/CK^−^/CD163^−^ TAMs in the intratumoral area was correlated with a favorable recurrence rate. These findings indicate that the specific subpopulation and localization of TAMs in the tumor microenvironment affect clinical outcomes in breast cancer. Overall, this study highlights the importance of TAMs and PD-L1 in breast cancer and suggests that they may be potential targets for therapy [11]. The purpose of the study of Seidel F et al. is to investigate the potential therapeutic value of complement inhibition in non-alcoholic steatohepatitis (NASH) and atherosclerosis. Specifically, the study aimed to assess the effects of anti-C5 antibody treatment on NASH and atherosclerosis in obese Ldlr^−/−^. Leiden mice were fed on a high-fat diet, and hepatic gene expression and pathway changes associated with the treatment were analyzed. The results of the study showed that anti-C5 treatment reduced the development of atherosclerosis but did not affect NASH. However, the treatment reduced the development of atherosclerosis, limiting the total lesion size and severity, independently of an effect on plasma cholesterol but with reductions in oxidized LDL (oxLDL) and the macrophage migration inhibitory factor (MIF). The study suggests that anti-C5 treatment in advanced stages of NASH is not sufficient to reduce the disease, while therapeutic intervention against established atherosclerosis could be beneficial to limit further progression [12]. The article by De Cunto et al. discusses the progression of chronic obstructive pulmonary disease (COPD) and the persistence of lung inflammation even after smoking cessation. The study was conducted in male C57 Bl/6 mice, where former smoker mice showed a reduced expression of histone deacetylases HDAC2 and SIRT1 and the marked expression of p-p38 MAPK and p-Ser10, which perpetuated inflammation and contributed to corticosteroid insensitivity. These mice also showed persistent lung neutrophilic influx and a high number of macrophages, which account for the intense staining in the alveolar structures of neutrophil elastase and MMP-9 and 8-OHdG. The release of “alarmins” from necrotic cells further perpetuates inflammation after smoking cessation. The study suggests that targeting specific molecular mechanisms associated with different phenotypes of the disease with a cocktail of drugs may provide better control of COPD in humans [13]. Pulmonary arterial hypertension (PAH) is a serious medical condition that has a high mortality rate despite the availability of various diagnostic and therapeutic options. While current treatments focus on vasodilation in the lungs, they do not address the underlying pathological changes that occur in the pulmonary vasculature. Therefore, there is a need for new therapeutic agents that can target pulmonary vascular remodeling. The review article by Martin de Miguel et al. aims to provide an overview of the molecular mechanisms that contribute to the development of PAH and the novel compounds being developed for its treatment. The article examines various cellular and molecular processes, including endothelial dysfunction, vascular smooth muscle cell proliferation, and inflammation, that contribute to the development and progression of PAH. The review also discusses the new pharmacological agents currently being developed to target these molecular mechanisms, such as tyrosine kinase inhibitors, peroxisome proliferator-activated receptor (PPAR) agonists, and soluble guanylate cyclase (sGC) stimulators. The article assesses the potential therapeutic value of these compounds in the management of PAH, including their mechanism of action, efficacy, and safety profile. The development of novel agents that can target pulmonary vascular remodeling may improve the prognosis of patients with PAH and provide a new treatment option for this challenging condition [14]. Atrial fibrillation (AF) is a common type of cardiac arrhythmia, and autoimmunity has been suggested to play a role in its development. Autoantibodies, which are produced by the immune system and can target the body’s tissues, may regulate heart’s rhythm and conduction and contribute to AF development. The study by Zygadło et al. summarizes current knowledge on the role of autoantibodies in AF, including their potential as prognostic and predictive markers for the disease. However, the exact role of different autoantibodies in AF is still a topic of ongoing debate, and the establishment of autoantibody profiles for different AF patient groups may be crucial for the development of novel treatment approaches. Further research is needed to better understand the mechanisms by which autoantibodies contribute to AF and how they may be targeted in the treatment of this disease [15]. The skin acts as a protective barrier against external stimuli, but chronic exposure to these factors can lead to local inflammation and tissue damage. Atopic dermatitis (AD) is a chronic inflammatory skin disease that is influenced by various environmental factors and involves various Th2 and proinflammatory cytokines. Recent studies suggest that cytokines released from atopic skin can also affect systemic organ function and contribute to the development of inflammatory diseases and malignancies. The review by Itamura et al. focuses on the relationship between AD and systemic inflammation and malignancies. It highlights the need for further research to better understand the mechanisms underlying the association between AD and systemic diseases, as well as the potential for novel therapeutic approaches targeting this connection [16]. Bladder cancer (BC) is prevalent cancer that requires a combination of invasive and non-invasive methods for diagnosis and surveillance. However, these methods have several limitations and are costly. Biomarkers such as proteins, genes, and RNAs have been extensively studied for BC diagnosis. Liquid biopsy, which involves analyzing cell-free DNA, non-coding RNA, and other subcellular structures, is emerging as an equally effective and less invasive approach. This review focuses on gene mutations, epigenetic modifications, and non-coding RNA molecules acquired by liquid biopsy. The review by Harsanyi et al. conducted an online search using various databases and set the sensitivity and specificity threshold to 80%. In the era of precision medicine, the development of laboratory techniques is driving the search for more sensitive and specific biomarkers for BC diagnosis, follow-up, and screening. The review concludes that future efforts should focus on validating the sensitivity, specificity, and predictive value of these biomarkers and their utility in clinical practice [17]. Long COVID-19 is a newly identified syndrome that develops after infection with SARS-CoV-2 and presents a wide range of clinical symptoms affecting multiple organs and tissues. The underlying molecular causes are related to immune dysregulation, persistent inflammation, epigenetic modifications, and altered neurotrophin release. Current prevention and management strategies for long COVID-19 are inadequate and require further research. Exercise is suggested as a valuable strategy for both the prevention and treatment of long COVID-19, as it can induce multiple positive effects on many organs simultaneously. Mind–body interventions such as Tai Chi, yoga, and meditation can also be integrated into exercise plans for stress management and immune system modulation. However, caution should be taken in employing exercise training protocols, as they may not be suitable for all patients. Patients’ physical capabilities and clinical features, as well as organ failure and comorbidities, should be considered before beginning any exercise program. Screening based on biomarkers modulated by exercise could be helpful for stratifying patients who will benefit from exercise and avoid significant side effects. The continuous monitoring of patients during training and avoiding overexertion exercises are necessary [18]. Moreover, SARS-CoV-2 research efforts have focused on understanding the biology of the virus and developing clinical applications for monitoring, detection, diagnosis, and treatment. Transcriptome analysis has contributed to generating new knowledge on viral sequences and intracellular signaling pathways that regulate the infection and pathogenesis of SARS-CoV-2. New approaches such as CRISPR-CAS, ASOs, and siRNA systems have emerged as promising strategies for battling the pandemic. RNA-based therapeutics have been used to develop clinical predictive models for SARS-CoV-2. Precision public health is essential in the management of patients infected with SARS-CoV-2. The fusion of transcriptomics, RNA-based therapeutics, and precision public health will allow the development of health systems that facilitate the acquisition of relevant clinical strategies for rapid decision making to assist in the management and treatment of the SARS-CoV-2-infected population to combat this global public health problem [19].
